# The new face of monkeypox virus: an emerging global emergency

**DOI:** 10.1016/j.nmni.2022.100989

**Published:** 2022-05-27

**Authors:** Nityanand Jain, Edouard Lansiaux, Raimonds Simanis

**Affiliations:** 1) Faculty of Medicine, Riga Stradiņš University, Dzirciema Street 16, LV-1007 Riga, Latvia; 2) Lille University School of Medicine, 2 Avenue Eugène Avinée, 59120 Loos, Lille, France; 3) Department of Infectiology, Faculty of Medicine, Riga Stradiņš University, LV-1007 Riga, Latvia

**Keywords:** Global, infections, monkeypox virus, pandemic, sexual transmission

More than a year ago in March 2021, over 19 senior experts and leaders in various fields from across the globe gathered virtually to participate in a tabletop simulative exercise on exploring the gaps in international biosecurity and pandemic response systems. The panel conducted by the non-profit Nuclear Threat Initiative (NTI; https://www.nti.org/) in association with Munich Security Conference (MSC; https://securityconference.org/), explored ways to tackle the effects of high-consequence biological threats like Covid-19 [1]. What makes this panel discussion remarkable was the pathogen that was simulated in the discussion. The group studied the global uncontrolled spread of a deadly and unusual strain of monkeypox virus (MPXV) that emerged in a fictional nation [1]. By the end of the simulated period of 18 months, the virus had infected 3 billion people whilst killing more than 270 million people worldwide [1].

Fast forward to today, May 2022, and the simulation seems to be becoming a reality. On 7th May 2022, the UK Health Security Agency (UKHSA) confirmed the first case of MPXV in a UK traveller who had made a round-trip to Nigeria [2]. In the following two weeks, more than 84 cases have been confirmed with an additional 61 suspected cases reported in 14 countries (Fig. 1) [3].

**Fig. 1:**
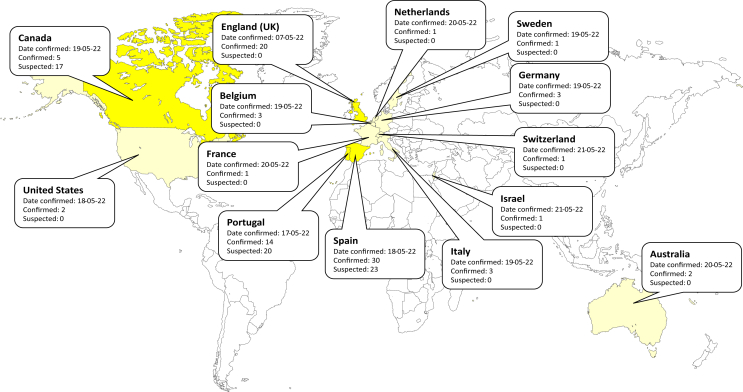
Map depicting the geographical spread of the Monkeypox Virus (MPXV) as of 21st May 2022. Date confirmed refers to the date of confirmation of the first MPXV case in the country. “Confirmed” refers to the number of confirmed cases whilst “Suspected” refers to the number of suspected cases in the respective countries. Note that the map is only for illustrative purposes and the authors remain neutral in regard to territorial disputes.

MPXV is one of the four human pathogenetic species belonging to the *Orthopoxvirus* genus in the *Poxviridae* family. Together with Variola virus (causative agent of now eradicated smallpox), Cowpox, and Vaccinia virus, these zoonotic viruses can infect a wide range of mammalian species [4,5]. Two epidemiologically distinct genetic clades of MPXV have been described in the literature – West African and Central African (or Congo Basin) clades. The West African clade has a lower-case mortality (3.6%) with no direct human-to-human transmission noted in comparison with the Central African clade with a higher case mortality (10.6%) and known human-to-human transmission [6].

MPXV is currently considered a rare zoonotic disease except in DRC (Democratic Republic of Congo) where the cases have grown exponentially. Since 1970, more than 35,000 cases have been documented (including confirmed, suspected, and probable) worldwide with >95% cases reported in DRC alone, followed by Nigeria and other neighbouring African countries [6]. Until 2003, the MPXV was not reported outside Africa, however, with an increase in trade, business, and travel, sporadic outbreaks have been reported across the globe, curiously all having their contact origins in Nigeria [6].

Previous reports have shown that outside Africa, the virus tends to predominantly infect adult males (>50% cases) in the age range of 10-21 years (Table 1) [6]. Symptoms develop in two phases – the invasion period and the skin eruption period. The invasion period (0-5 days post contact) presents with fever, headache, muscle aches, fatigue, backache, and chills. Lymphadenopathy during this phase is characteristic of MPXV infection [7]. Development of rash starts 1-3 days after the fever and usually affects the face, palms, soles, oral and genital mucous membranes, and conjunctiva. Rash progresses sequentially through macules, papules, vesicles, pustules, followed by crusts that dry up and fall off [7]. Though not fatal, the symptoms are self-limiting lasting from 2 to 4 weeks.

**Table 1:** Characteristics of confirmed and suspected MPXV cases as of 21st May 2022

Characteristics	No. of cases	Characteristics	No. of cases
**Case Status**		**Hospitalization**	
Confirmed	86 (59%)	Yes	21 (14%)
Suspected	61 (41%)	No	37 (25%)
		Unknown	89 (61%)
**Age Groups**			
20-40	56 (38%)	**Case Isolation**	
40-60	2 (1%)	Yes	59 (40%)
Unknown	89 (61%)	Unknown	88 (60%)
**Gender**		**Travel History**	
Male	106 (72%)	Yes	12
Female	1 (1%)	No	42
Unknown	40 (27%)	Unknown	93
**Reported Symptoms**		**Source of Contact**	
Fever	19	Sexual Contact	
Rash (vesicular and/or unspecified)	8	Suspected sexual contact	28 (19%)
Genital and oral ulcers	22	Man-to-man sex	4 (3%)
Lesions (ulcerative, skin)	21	Sauna	15 (10%)
Swallowing difficulties	1	Unknown	100 (68%)
Perianal papules	1		
Inguinal adenopathy	1		
Unknown	94		

Based on the mathematical modelling of available literature, it has been predicted that MPX may exist in a semi-endemic equilibrium, where there is no infection in its animal host, but the disease persists in humans [8]. Though the authors didn't find viral fitness to become endemic solely through human transmission, mutations in viral proteins could upset the model [8]. Studies have elaborated and highlighted the probable factors that could lead to a rampant spread of the MPX virus across the globe (Fig. 2) [1,4,6]. Addressing these issues is of utmost priority to prevent another pandemic, especially since the disastrous effects of Covid-19 are still strongly present in the collective human memory.

**Fig. 2:**
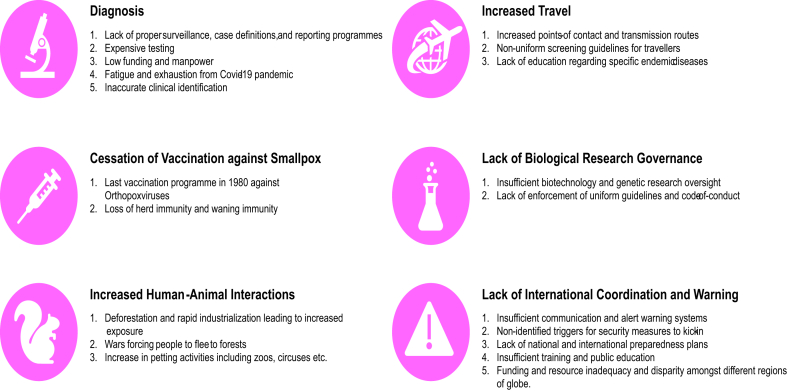
Factors promoting the rapid spread of the MPX virus that needs to be addressed to prevent rampant spread of the virus and further epidemiological escalation of the situation.

A group of researchers from Portugal has open-sourced the first draft of the genomic sequence of the MPXV, which reveals the virus belonging to the West African clade with close links to the viruses which were previously reported in the UK, Israel, and Singapore in 2018-19 [9]. Noticeably, according to the European Centre for Disease Prevention and Control (ECDC), “This is the first time that chains of transmission are reported in Europe without known epidemiological links to West or Central Africa, where this disease is endemic” [10].

What is even more intriguing is that this is also the first time worldwide whereby cases are being reported amongst the men who have sex with men (MSM) community [10]. This is evident also from the large number of cases reporting genital and oral ulcers (Table 1). Close contact has been implicated in the transmission of the virus however, the identification of the Western African clade as the causative agent is a puzzling mystery.

Human sexual transmission of *Orthopoxvirus* has been documented previously [11,12]. However, most cases have been sporadic isolated instances where an unvaccinated partner got infected post-coitus from a vaccinated partner (vaccinated against Vaccinia virus) having an unhealed vaccination site. In fact, a few years ago, a case of secondary and tertiary transmission of the Vaccinia virus in a MSM patient was reported by the US Centre for Disease Control and Prevention (US CDC) [13].

Whilst contact tracing and the source of spread are still under investigation, Spanish authorities revealed that all 23 men with suspicion of infection had visited the same Paraiso sauna, a gay-friendly establishment, that has since been closed (Table 1) [14]. Similarly, Australian authorities have issued an alert for passengers on the two flights that were taken by the confirmed patient from London to Melbourne [15]. Interestingly, the Australian confirmed case was diagnosed by an astute general practitioner (GP) [15], which highlights the importance of GPs in early detection, contact tracing, and breaking of transmission chains.

Moving forward, rapid response and action are needed from all parties including society, healthcare professionals, and policymakers. A detailed interpretation of this topic has been summarized by Giulio and Eckburg [16]. Vaccination against smallpox has shown to have an efficacy of around 85% against MPXV [17], however, mass vaccination, as with SARS-CoV-2 would be counterproductive in this case. A more targeted “ring vaccination” approach can be recommended where high-risk close contacts only are vaccinated [18]. Other newer therapeutical options including the Modified Vaccinia Ankara vaccine against MPXV, and the recently approved antiviral Tecovirimat may be used in limited clinical settings with careful observation. Vaccinia IVIG (VIVIG) could also be used for the management of complications and prevention of the development of long-term sequelae.

Finally, the lessons learned during the Covid-19 pandemic should not be forgotten, rather should be adjusted, and rapidly implemented. As author Donna Maltz summarizes in her book “Conscious Cures: Solutions to 21st Century Pandemics” - *“COVID-19 slowed us down, but what have we learned? The virus gave us time to think, but what are we thinking?”* In the context of MPXV, these questions are for all of us to consider before it’s too late.

## Ethical approval

Not applicable. All data presented in the study has been collected from open-source platforms with proper citation and/or from media sources.

## Acknowledgments

The support of ECOMSIR (European Collaboration of Medical Students in Research) and Riga Stradiņš University is greatly acknowledged.

## Conflicts of interest

The authors declare no conflicts of interest in regards with the present editorial. The viewpoints presented are personal and doesn't necessarily reflect the viewpoints of the affiliated institutions.

## Funding

The present editorial didn't receive any external funding and was self-supported by the authors.

## Author contributions

NJ and EL conceptualized the present editorial, whilst all authors were involved in data collection and preparation of the manuscript**.** Supervision was done by RS. Visualizations was done by NJ and EL. All authors have read and agreed to the final version of the editorial for publication.

## References

[1] Yassif J.M., O’Prey K.P., Issac C.R. Strengthening global systems to prevent and respond to high-consequence biological threats: results from the 2021 tabletop exercise conducted in partnership with the Munich security conference. https://www.nti.org/wp-content/uploads/2021/11/NTI_Paper_BIO-TTX_Final.pdf.

[2] World Health Organization (WHO) Monkeypox - United Kingdom of great Britain and Northern Ireland. https://www.who.int/emergencies/disease-outbreak-news/item/2022-DON381.

[3] Kraemer M., Global.health Monkeypox Twitter post. https://twitter.com/MOUGK/status/1527055553876348928?s=20&t=_bXBbMFBDKmR6drFjGfZZw.

[4] Sklenovská N., Van Ranst M. (2018 Sep 4). Emergence of monkeypox as the most important orthopoxvirus infection in humans. Front Public Health.

[5] Shchelkunov S.N., Marennikova S.S., Moyer R.W. (2005).

[6] Bunge E.M., Hoet B., Chen L., Lienert F., Weidenthaler H., Baer L.R., Steffen R. (2022 Feb 11). The changing epidemiology of human monkeypox-A potential threat? A systematic review. PLoS Negl Trop Dis.

[7] World Health Organization (WHO) Monkeypox. https://www.who.int/news-room/fact-sheets/detail/monkeypox.

[8] Bankuru S.V., Kossol S., Hou W., Mahmoudi P., Rychtář J., Taylor D. (2020 Jun 22). A game-theoretic model of Monkeypox to assess vaccination strategies. PeerJ.

[9] Isidro J., Borges V., Pinto M., Ferreira R., Sobral D., Nunes A. (May 2022). First draft genome sequence of Monkeypox virus associated with the suspected multi-country outbreak. https://virological.org/t/first-draft-genome-sequence-of-monkeypox-virus-associated-with-the-suspected-multi-country-outbreak-may-2022-confirmed-case-in-portugal/799.

[10] European Centre for Disease Prevention and Control (ECDC) Epidemiological update: Monkeypox outbreak. https://www.ecdc.europa.eu/en/news-events/epidemiological-update-monkeypox-outbreak.

[11] Said M.A., Haile C., Palabindala V., Barker N., Myers R., Thompson R. (2013 Dec). Transmission of vaccinia virus, possibly through sexual contact, to a woman at high risk for adverse complications. Mil Med.

[12] Centers for Disease Control and Prevention (CDC) (2007 May 4). Vulvar vaccinia infection after sexual contact with a military smallpox vaccinee--Alaska, 2006. MMWR Morb Mortal Wkly Rep.

[13] Centers for Disease Control and Prevention (CDC) (2013 Mar 1). Secondary and tertiary transmission of vaccinia virus after sexual contact with a smallpox vaccinee--San Diego, California, 2012. MMWR Morb Mortal Wkly Rep.

[14] Times of India Spanish monkeypox outbreak linked to sauna. https://timesofindia.indiatimes.com/world/europe/spanish-monkeypox-outbreak-linked-to-sauna/articleshow/91706597.cms.

[15] 7 news.com.au Australia’s first confirmed monkeypox case recorded in Victoria. https://7news.com.au/news/public-health/warning-in-vic-for-rare-monkeypox-disease-c-6867171.

[16] Di Giulio D.B., Eckburg P.B. (2004 Jan). Human monkeypox: an emerging zoonosis. Lancet Infect Dis.

[17] Eteng W.E., Mandra A., Doty J., Yinka-Ogunleye A., Aruna S., Reynolds M.G. (2018 Sep 21). Notes from the field: responding to an outbreak of monkeypox using the one health approach - Nigeria, 2017-2018. MMWR Morb Mortal Wkly Rep.

[18] Kozlov M. (2022 May 20). Monkeypox goes global: why scientists are on alert. Nature.

